# Pain cues override identity cues in baby cries

**DOI:** 10.1016/j.isci.2024.110375

**Published:** 2024-06-24

**Authors:** Siloé Corvin, Camille Fauchon, Hugues Patural, Roland Peyron, David Reby, Frédéric Theunissen, Nicolas Mathevon

**Affiliations:** 1 ENES Bioacoustics Research Lab, CRNL, University of Saint-Etienne, CNRS, Inserm, Saint-Etienne, France; 2 Université Jean-Monnet-Saint-Etienne, INSERM, CNRS, UCBL, CRNL U1028, NeuroPain team, 42023 Saint-Etienne, France; 3 Université Clermont Auvergne, CHU de Clermont-Ferrand, Inserm, Neuro-Dol, Clermont-Ferrand, France; 4 Neonatal and Pediatric Intensive Care Unit, SAINBIOSE laboratory, Inserm, University Hospital of Saint-Etienne, University of Saint-Etienne, Saint-Etienne, France; 5 Institut universitaire de France, Paris, France; 6 Helen Wills Neuroscience Institute, University of California, Berkeley, Berkeley, CA 94720, USA; 7 Department of Psychology, University of California, Berkeley, Berkeley, CA 94720, USA; 8 Department of Integrative Biology, University of California, Berkeley, Berkeley, CA 94720, USA; 9 Ecole Pratique des Hautes Etudes, CHArt lab, PSL University, Paris, France

**Keywords:** Natural sciences, Behavioral neuroscience, Social sciences, Research methodology social sciences

## Abstract

Baby cries can convey both static information related to individual identity and dynamic information related to the baby’s emotional and physiological state. How do these dimensions interact? Are they transmitted independently, or do they compete against one another? Here we show that the universal acoustic expression of pain in distress cries overrides individual differences at the expense of identity signaling. Our acoustic analysis show that pain cries, compared with discomfort cries, are characterized by a more unstable source, thus interfering with the production of identity cues. Machine learning analyses and psychoacoustic experiments reveal that while the baby’s identity remains encoded in pain cries, it is considerably weaker than in discomfort cries. Our results are consistent with the prediction that the costs of failing to signal distress outweigh the cost of weakening cues to identity.

## Highlights

• Pain cries have a more variable pitch than discomfort cries
• Individual signatures in pain cries are less reliable than in discomfort cries
• The acoustic space of pain cries is shrunk compared to discomfort cries
• Generalizing baby identification from discomfort to pain cries is challenging

## Graphical abstract

**Figure:**
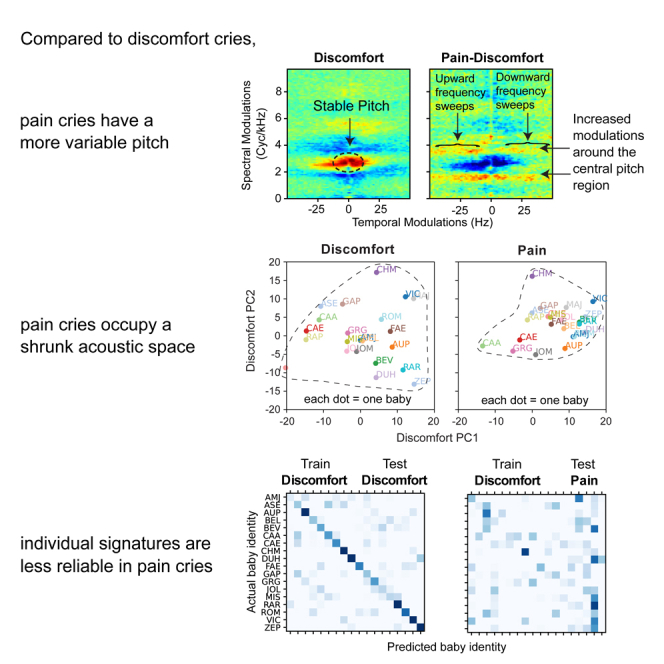

## Introduction

Babies’ cries soliciting parental care are generally triggered by pain, discomfort, hunger, or separation from parents or other caregivers.^1^ While some of the information conveyed by these cries is fairly stable over time and linked to the idiosyncratic characteristics of the sender, such as their age, size, weight, and the anatomy of their vocal tract (“static information” or “vocal signature” allowing a given baby to be identified), other information is labile and relates to the current emotional and physiological state (“dynamic information” signaling the baby’s immediate needs).^2^ Parents or other caregivers adjust the quantity and quality of care provided to their baby according to this information.^3^^,^^4^ Previous studies have shown that they also quickly learn to identify their baby by their cries, become better at detecting dynamic variations in the cries, and are more accurate in judging their baby’s current emotional and physiological state than those of other babies.^5^

Studies with animals have examined the constancy of an individual vocal signature between different vocalizations. The results vary from species to species. In zebra finches (*Taeniopygia guttata*), for example, whose vocal repertoire includes many different call types emitted in various contexts and with various functions, each call has characteristics that allow to identify the individual caller.^6^ However, these individual vocal signatures are not transferable between call types, and it is not possible to identify an individual from one type of call by using the acoustic criteria for individuality found in another type of call. A given individual therefore has as many vocal signatures as call types in its repertoire. In mammal species such as the red deer (*Cervus elaphus*) or the cow (*Bos taurus*), the coding of static information containing the identity of the individual is shared across different vocalizations bearing different dynamic information,^7^ such as vocalizations with positive and negative valence.^8^ In these species, variations in dynamic information have little effect on static information, which remains stable. In other species, the reliability of the individual vocal signature can vary between vocalizations. This is the case for the bonobo’s (*Pan paniscus*) vocal repertoire, where calls emitted in contexts of high-arousal are more individualized than vocalizations emitted in contexts of low arousal.^9^ In the bonobo, the acoustic gradation that codes for dynamic information (i.e., the individual’s excitation level) is accompanied by a variation in the static information that supports vocal individuality.

In humans, the fundamental frequency of the adult voice (pitch) is a reliable marker of individuality, which is preserved in verbal and nonverbal vocalizations.^10^ It is known that the fundamental frequency varies predictably during the development of an individual and stabilizes in adulthood.^11^^,^^12^ However, little is known about how static information and dynamic information interfere in human vocalizations. In the case of infant cries, a reliable individual vocal signature is present in discomfort cries (emitted during bathing^13^^,^^14^ or when the baby is hungry^15^^,^^16^^,^^17^), but it is not known whether this signature is also found in distress cries provoked by pain. One hypothesis is that a signature common to all of a baby’s cries would make it easier to identify the baby in all circumstances, increasing the motivation of the parent or any other caregiver to react to never previously heard cries emitted by their baby. An alternative hypothesis is that by making the baby less identifiable, a difference in individual signature—or a reduction in its reliability—in their pain cries would increase the probability of engaging the response of any nearby adult, a strategy that could be well suited to emergency situations.

Here we test these hypotheses by examining whether the static information that enables a baby to be identified by their cries recorded in a discomfort context is preserved in cries elicited by a pain context. We first quantify the reliability of this information in both emission contexts using classifiers trained either with discrete acoustic parameters or with modulation power spectra (MPS). We then test the reliability of classifiers trained on one type of cry (discomfort or pain) in identifying babies from the other type of cry. Finally, we conduct psycho-acoustic experiments to assess the ability of adult listeners—women and men, parents and non-parents—to recognize an assigned baby from their pain cries after familiarization based on their discomfort cries.

## Results

### Pain cries have a more variable pitch than discomfort cries

**Figure 1:**
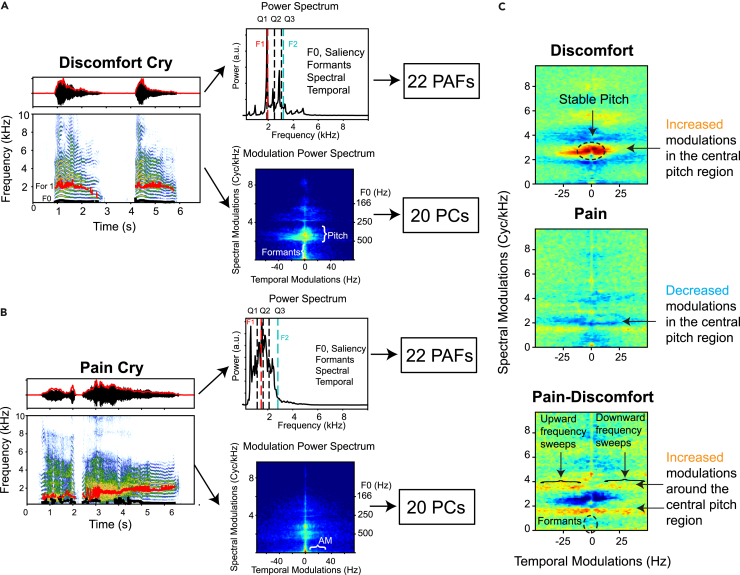
Pain cries have more variable pitch than discomfort cries (A and B) Example of cries from one baby in the discomfort (A) and pain (B) conditions and extraction of acoustical features. *Left panels*: the vocalization is depicted as an oscillogram (top) and spectrogram (bottom). In the oscillogram, the sound pressure waveform is shown as a black line and its amplitude envelope with a red line. The time-varying fundamental and time-varying first formant are shown on the spectrogram with black and red lines, respectively. *Right panels*: a total of 22 predefined acoustic features (PAFs) are extracted from the measures obtained from the amplitude envelope, the power spectrum, and the time-varying fundamental and formants. The modulation power spectrum (MPS) is obtained by calculating and averaging the amplitude of the 2D Fourier transform of spectrogram in 1s windows. The first 20 principal components (PCs) of the MPS are also extracted as acoustical features. (C) Average MPS for discomfort, pain, and the difference between the pain and discomfort conditions. The average difference is obtained from pairwise differences in log power (dB) of the average MPS in the pain condition minus the log power of the average MPS in the discomfort condition. The overall pairwise average is obtained from the 22 babies in the acoustic database. In the pain condition, the modulations that are associated with pitch (and pitch quality) are greatly reduced (blue region around 3 cyc/kHz), whereas the spectral modulations around this central pitch region are enhanced (red regions).

We recorded each baby (*N* = 22; 2 months old) under two conditions^2^: during bathing, undressing or dressing by the parents at the baby’s home (“discomfort cries”) and during a vaccination session at the doctor’s office (“pain cries”). We isolated 284 cry sequences from these raw recordings (4 sequences of pain cries and between 2 and 19 sequences of discomfort cries per baby; average duration of sequences = 6.3 ± 1.1 s), and described their acoustic structure using 22 predefined acoustic features (PAFs, characterizing the amplitude envelope, power spectrum, fundamental frequency—pitch—and formants), and the MPS^18^ (characterizing the power of the signal as a function of temporal and spectral modulations; see STAR Methods; Figures 1A and 1B).

While the mean pitch frequency is not significantly different between discomfort and pain cries (mixed-effects linear model, Wald test z = 1.634; *p* = 0.102), the MPS reveals that discomfort cries have a more stable pitch than pain cries whose pitch extends over a wider range and is more modulated (Figure 1C, discomfort panel: the central region in red near 3 cyc/KHz indicates a pitch around 330 Hz; Figure 1C, pain panel: the red regions around the 3 cyc/kHz blue region highlight that the range of pitches increased from ∼250 to 580 Hz, with corresponding additional increases in rapid frequency modulation power for those extreme values of pitch −20 to 20 Hz). Pain cries are also marked by wider amplitude modulations (Figure 1C, pain panel: temporal modulations power along the x axis, i.e., with 0 cyc/kHz). In addition, pitch saliency, as quantified by the temporal regularity of the signal (see STAR Methods), is significantly lower in pain cries, meaning that they are on average more noisy and less tonal than discomfort cries (mixed-effects linear model, dSal = −0.1 ± 0.06 [2SE]; z = −3.364; *p* = 0.001).

### Individual signatures in discomfort cries do not generalize to pain cries and pain cries signatures are less reliable

**Figure 2:**
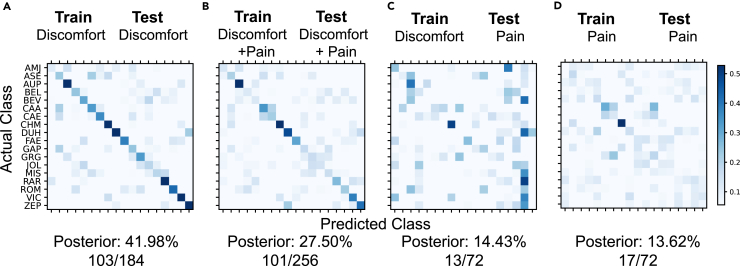
Individual signatures are less reliable in pain cries than in discomfort cries Linear discriminant analysis (LDA) based on the MPS was used to obtain confusion matrices showing the posterior conditional probability of correct baby identity prediction obtained in cross validation. Eighteen babies for which we had at least 5 samples of discomfort cries for the training set were used. The classifier was either trained and tested with discomfort cries only (A), with both discomfort and pain cries (B), trained on discomfort cries then tested on pain cries (C), or train and tested with pain cries (D). The posterior shown at the bottom of the graphs are the average of the diagonal for the corresponding confusion matrix (chance level = 1/18 = 5.6%). The numbers below correspond to the number of baby cries in the cross-validation sets that were correctly classified using the maximum posterior relative to the total size of the validation set.

We quantified the possibility of identifying babies from their cries using cross-validated supervised classifications with machine learning algorithms. The classifiers were tested and trained with the modulation power apectra (MPS). The MPS was chosen as a feature space because it captures all of the joint spectral and temporal structures present in the sound signals without making *a priori* assumptions on what constitutes informative features. Contrary to spectrograms, MPS across exemplars for particular conditions (e.g., baby in discomfort) can be averaged.^18^ Figure 2 shows the confusion matrices illustrating the correct classification probabilities obtained with linear discriminant analyses. The cries were correctly attributed to the emitting babies in 42.0 ± 7.3% (mean posterior ± 2SE) of cases when the classifier was trained with discomfort cries and then tested with different cries but recorded in the same discomfort context (Figure 2A; cries from 18 babies for which we had at least 6 samples of discomfort cries; chance level = 5.6%; see STAR Methods for details). This result confirms the presence of an individual signature in discomfort cries. When the classifier was trained and then tested with a mix of discomfort and pain cries, the reliability of the classification decreased (mean posterior ± 2SE = 27.5 ± 5.6%; Figure 2B). Thus, the identity signature might be distinct in discomfort versus pain cries and/or pain cries carry less individual information. To assess the relative strength of these two factors for the decreased performance in the mixed condition, we performed two additional classifications. First, we trained with discomfort cries and tested its performance on pain cries: when forced to generalize across conditions, the identification of became much weaker (mean posterior ± 2SE = 14.4 ± 8.3%; Figure 2C). Second, we trained and tested the classifier using only pain cries: the performance of the classifier was similarly just as poor (mean posterior ± 2SE = 13.6 ± 8.1%; Figure 2D). The statistical significance in the decrease in performance was assessed by examining the number of correctly classified babies in leave one out cross validation for within context and in simple cross-validation across context. In the discomfort context, the identity of 103/184 (56%) baby cries were correctly classified. In the mixed context, 101/256 (39%) were correctly classified; significantly less than in the discomfort context (*p* = 0.00069, Fisher exact test for independence). For a classifier trained on discomfort and tested on pain cries, the identity of only 13/72 (18%) was correctly classified, significantly less than both the discomfort context (*p* = 2.8 10^−8^) and the mixed context (*p* = 0.00070). Finally, for the classifier trained and tested on pain cries, the identity of 17/72 (23%) was correctly classified, significantly less than both the discomfort context (*p* = 3.2 10^−6^) and the mixed context (*p* = 0.01768) but not significantly different from the cross-condition (*p* = 0.53). Thus, pain cries do carry less individual information than baby cries, which could explain for the large part the lack of generalization across conditions. But note also that the confusion matrices in C and D are quite different with the disorganization shown in C showing poor use of identity cues obtained by training in the discomfort set. Nonetheless, for all four combinations of training and test datasets, the level of correct classification was significantly higher than chance (binomial test, *p* < 0.01; see details in Table S1), highlighting that pain cries nevertheless retain a small amount of information about the baby’s identity. The performance of other classifiers (quadratic discriminant analysis [QDA] and random forest [RF]) gave similar results (Table S1).

To specify the acoustic features carrying the individual signatures, we then trained new LDA, QDA, and RF classifiers using either the 22 PAFs or a subset of 10 PAFs retaining only the spectral features of the cries (see STAR Methods for a list of parameters). These approaches using the PAFs gave lower classification values than those using the MPSs, which is not surprising since the MPSs represent the spectro-temporal structure of the signal more holistically than a set of discrete acoustic parameters (see Figure S1 and Table S2). However, similar to what we obtained with the MPSs, the performance of the classifier using the 22 PAFs and trained with discomfort cries dropped from 31.9% when tested with other discomfort cries to 14.2% when tested with pain cries (*p* = 5.16 10^−7^, Fisher exact test for independence). The performance for a classifier trained and tested on pain cries as 14.9% also significantly less than the 31.9% obtained for discomfort (*p* = 0.0042) but similar to the performance of the cross-condition of 14.2% (*p* = 0.0864). These performances are similar to those obtained by the classifier using the 10 selected PAFs, suggesting that the most salient acoustic features characterizing the information for baby identity are contained in the spectral shape of the cry, its tonality (pitch salience), and the fundamental (pitch) and its modulations (30.5% of correct classification when tested on discomfort cries using the 10 selected PAFs; see Figure S1 and Table S2).

### The acoustic space of pain cries is shrunk compared to discomfort cries

**Figure 3:**
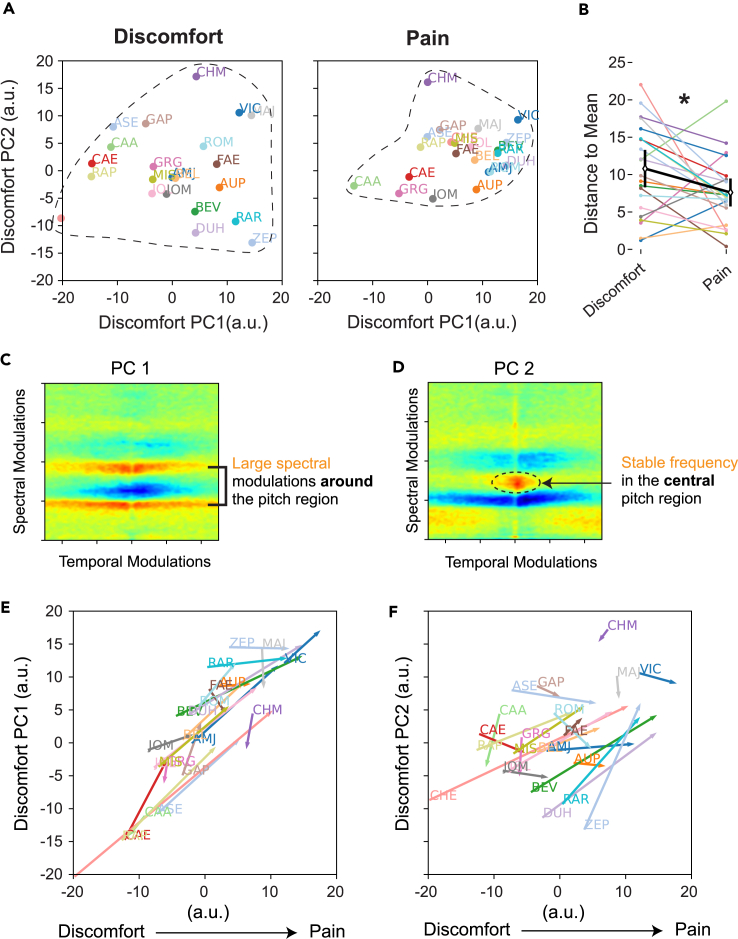
The acoustic space of pain cries is shrunk compared to discomfort cries (A) Individual signatures in the acoustic space of discomfort cries are degraded in pain cries. The average modulation power spectrum (MPS) for each baby is plotted in a 2D acoustic space defined by the first two principal component coefficients (PC1 and PC2) of discomfort cries, in the discomfort and pain condition. The pain condition results in a shift of PC1 toward higher values as well as a reduction of the acoustic space spanned (dotted circles). (B) The distance between the average position of each baby’s cries and the centroid decreased significantly in the pain condition. This shrinking is not observed when PC is obtained from pain cries (see Figure S2). (C and D) The two first principal components PC1 and PC2 derived from the MPS. (E) Trajectories in acoustic space of each baby cry from average discomfort (base of arrow) to average pain (arrowhead). The x axis is the single discriminant function in the MPS that best separates cries obtained in the discomfort condition from cries obtained in the pain condition. The y axis are the positions in the first principal component obtained from the average MPS per baby in the discomfort condition. The first acoustic dimension that best discriminates the identity of the babies in the bath condition is very similar to the best acoustic dimension that separates discomfort from pain cries. (F) A large fraction of the information available along discriminant dimension for identifying the baby identity in the discomfort condition is lost in the pain condition. Similar representation of each baby cries in the acoustic space as 3E, the y axis being the positions in the second principal components derived from the MPS. The spread of the data points is reduced as one goes from discomfort to pain.

Figure 3A shows the average positions of each baby’s discomfort cries (Figure 3A, left) and pain cries (Figure 3A, right) in a two-dimensional space defined by the first two principal components (PCs) of the MPS in the discomfort condition (these two PCs capture 55% of the variability between discomfort cries, and are equivalent to linear discriminant functions describing the best acoustical subspace for discriminating baby identity using discomfort cries; see STAR Methods for details of this MPS dimensional reduction). As can be seen by comparing the distribution of discomfort cries and pain cries (respectively, the left and right panels of Figure 3A), the pain cries occupy a smaller area in the acoustic space than the discomfort cries. This observation is consistent with the result of the classifiers we reported earlier: it is more difficult to discriminate between babies based on their cries of pain when one is basing its individual signature in an acoustic space describing discomfort cries.

We quantified this contraction of the acoustic crying space in the pain condition by calculating the distance between the average position of each baby’s cries in the 2D acoustic space and the centroid (average position) of all the babies’ cries. As shown in Figure 3B, this distance decreases significantly in the pain condition for the vast majority of babies, which highlights the greater homogeneity of pain cries between babies (mean distance in the discomfort condition = 10.80 ± 2.50 [2SE]; mean distance in the pain condition = 7.61 ± 1.87 [2SE]; paired t test D = 3.19; t(21) = 2.251; *p* = 0.0352).

To test whether pain cries could still encode an individual signature but along acoustic dimensions distinct from those obtained from discomfort cries, we repeated the previous analysis in a space where the two dimensions corresponded to the first two PCs of the MPS of pain cries instead of discomfort cries (see Figure S2). While this approach optimized the discrimination of babies recorded in the pain condition, we did not observe any significant variation in the occupation of the acoustic space between the discomfort and pain conditions (mean distance to the centroid in the discomfort condition = 8.30 ± 1.73; mean distance to the centroid in the pain condition = 9.77 ± 2.13; t(21) = −1.141; *p* = 0.2666). Thus, we do not find a separate set of acoustical dimensions that would capture individual signature in pain cries beyond one might deduce from the acoustical space spanned by discomfort cries.

### The main acoustic dimension carrying individuality is similar to the one separating discomfort cries from pain cries

Figures 3C and 3D show how the two PCs PC1 and PC2 derived from the MPS capture the variability of discomfort cries between different babies. The first PC (PC1, Figure 3C) is characterized by broad spectral modulations framing the pitch. This means that inter-individual differences between cries are particularly explained by variations in pitch modulation (i.e., some babies produce cries whose pitch varies little over time, while others modulate the pitch of their cries more strongly). As we reported in the first part of the results, variations in pitch modulation are also an essential dimension of the difference between discomfort and pain cries. Figure 3E illustrates this correlation between the PC1 dimension separating individual babies (y axis) and the dimension separating discomfort cries from pain cries (x axis). On the figure, each baby is represented by an arrow, the base of which indicates the average position of its discomfort cries and the tip the average position of its pain cries. The arrows are essentially oriented along the diagonal axis of the graph, highlighting a strong correlation between the two axes of the graph (correlation between the x and y coordinates of the discomfort cries: r = 0.899, Fisher’s exact test *p* = 1.295e-08; correlation for the pain cries: r = 0.903, *p* = 8.933e-09; correlation for pairwise difference discomfort-pain: r = 0.857, *p* = 3.438e-07, *N* = 22 babies). It is therefore remarkable that the coding of individual identity along PC1 is to a good extent along the same dimension as the coding of the discomfort versus pain context.

The second PC (PC2, Figure 3D) that best discriminates discomfort cries between babies is characterized by positive weights in the central region of the pitch (red region) and by an inhibitory band (blue) capturing a reduction in spectral modulations. The babies that score high on PC2 exhibit a strong stable pitch percept (low temporal modulations in pitch). In contrast, those that score low will either have a distinctly lower or higher mean fundamental frequency and/or more variability in pitch modulations. As shown in Figure 3F, there is also some correlation between the PC2 dimension separating individual babies (y axis) and the dimension separating discomfort cries from pain cries (x axis) but it is much weaker (correlation between the x and y coordinates of the discomfort cries: r = 0.382 *p* = 0.07913; correlation for the pain cries: r = 0.468 *p* = 0.02791, correlation for pairwise differences: r = 0.727, *p* = 0.0001). In addition, although the distribution of the orientation of the arrows indicating the trajectories followed by each baby from their discomfort cries to their pain cries is more random than for PC1, it nevertheless shows a reduction in the dispersion of cries when moving from the discomfort context to the pain context (distance from the mean for the discomfort condition = 6.03 ± 1.93 [2SE]; for the pain condition = 3.75 ± 1.30 [2SE]; difference = 2.280; t(21) = 2.282; *p* = 0.033). Much of the information available along this second dimension to discriminate babies in the discomfort condition is thus lost in the pain condition.

To further illustrate the fact that this analysis and visualization captures the shift (PC1) and shrinking (PC1 and PC2) of the acoustic space spanned by the individual signatures of baby cries, we can examine the cries of baby VIC. VIC’s discomfort and pain cries are acoustically close to each other and are found close to the centroid of the pain cries of all babies (see Figures 3E and 3F). It is therefore not surprising to see that our LDA classifier trained on discomfort cries but tested on pain cries systematically misassigned pain cries to VIC (see Figure 2C). In our psychoacoustical test with human listeners (see the following text), we also found that subjects that were assigned VIC as the “familiar” baby and trained on its discomfort cries, were better at discriminating its pain cry than subjects trained with other babies (71.9% correct vs. 39.2%). Finally, we found similar findings with PCs based on the 22 PAFs instead of the MPS, but with the order of PCs flipped. With PAFs, the values on the second PC correlate with scores along the dimension that best separates discomfort from pain cries. The magnitude of the PC2 weightings shows that this second PC principally captures changes in the coefficient of variation of the fundamental, the max fundamental and the pitch saliency. As we discussed previously, when interpreting the differences in MPS, relative to cries in the discomfort condition, cries in the pain condition show higher variations in fundamental, higher max fundamental and lower saliency (see Figure S3).

### Generalizing familiar baby identification from discomfort to pain cries is challenging for human listeners

We tested whether adult listeners are able to generalize a learned individual signature present in discomfort cries to extract information about a baby’s identity from their pain cries. Participants (*N* = 100) first listened to discomfort cries from a “familiar” baby (two training sessions one day apart, 5 different cries from the same baby per session, different cries between sessions). A few hours later (mean delay = 6.4 ± 4.4 h, min–max = 3–22.6), the participants listened to 20 different pain cries, including 4 cries from their “familiar” baby and 16 cries from unknown babies (2 boys and 2 girls; see STAR Methods for details), which they had to classify as either “familiar” baby or “unknown” baby.

**Figure 4:**
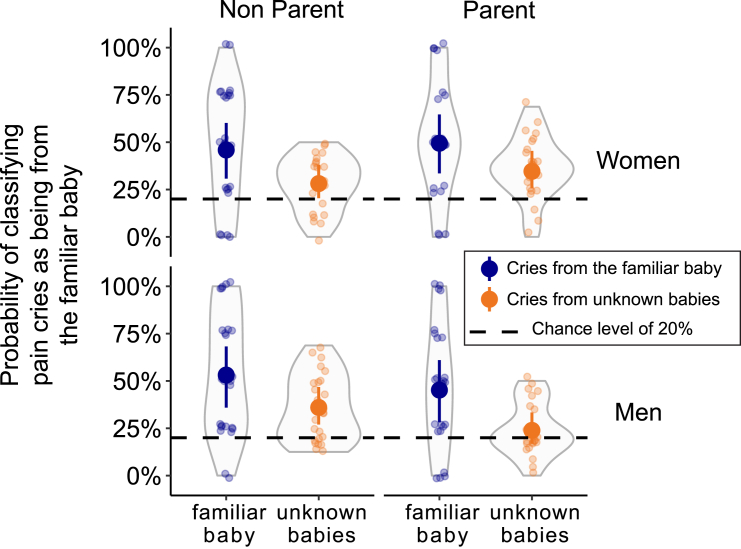
Generalizing familiar baby identification from discomfort to pain cries is challenging for human listeners Results of the playback test. Circles represent the individual data and solid disks the medians of the posteriors distributions, with bars indicating the 95% CI. The dotted lines indicate the 20% chance level. Subjects were able to recognize a familiar baby from pain cries when they had previously listened only to discomfort cries, but with a high false-positive rate. The ability to identify a familiar baby from pain cries was independent of the listener’s sex and parenting experience.

The tested listeners were able to identify their familiar baby from their pain cries significantly better than chance (Bayesian mixed model, median of the posterior for correct recognition 48.5%, 95% CI [35.1, 59.8], chance level of 4/20 = 20%; Figure 4). Despite a relatively high false positive rate (cries from unknown babies considered to be from the familiar baby; 30.8% (95% CI [24.3, 38.8]), familiar babies were 17.7% more likely to be identified than unknown babies (95% CI [1.7, 30.6]). We found no significant difference in this ability to recognize the familiar baby between women and men (−1.4% [-13.3, 11.3]), nor between parents and non-parents (−2.2% [−14.5, 11.4], Figure 4). Mothers and non-parent men had a higher probability of false-positive errors than fathers, with 10.6% (1.7, 19.5) and 12.1% (2.4, 21.6) more errors, respectively.

To compare the ability of human listeners with that of a machine learning algorithm, we trained an LDA classifier with the MPS by faithfully reproducing the experimental design used with humans (i.e., 10 training cries per familiar baby and 20 cries of pain—4 from the familiar baby and 16 from unknown babies—for the test phase). The performance obtained with the classifier was similar to that of humans (mean ± 2SE: classifier 52.1 ± 5.1% versus posteriors for human recognition 52.6 ± 6.0%).

## Discussion

This study shows that the individual acoustic signature carried by human baby cries emitted in a context of simple discomfort is only partly preserved in cries recorded in a painful context. Not only are pain cries less individually signed than discomfort cries but the information on individual identity that both classifiers and human subjects could obtain from the much more frequent discomfort cries does not generalize to pain cries. It is therefore significantly more challenging to identify a given baby by its pain cries than its discomfort cries.

The presence of an individual vocal signature in discomfort cries is an established fact, and can be explained by idiosyncratic characteristics of both the sound source (values of the fundamental frequency of the cry) and the vocal tract (length and shape of the tract).^14^ Previous studies have thus shown that mothers recognize their own babies by their cries of discomfort.^15^^,^^16^ This ability is acquired through exposure to the baby in the first few days after birth.^13^ If they care for their baby, fathers are as good as mothers at identifying them by their cries,^14^ and non-parents can identify an assigned baby after a short exposure.^13^ In the present study, we again demonstrated the importance of learning to identify a baby using their cries. Intriguingly, our results highlight a convergence of these individual signatures in the acoustic space of cries when looking at pain cries. This suggests that babies share, to some extent at least, the same ways of expressing pain through their cries. This “universal coding” of pain comes at the expense of the coding of individual identity.

The fact that the PC1 that best characterizes the individual signatures carried by discomfort cries in the MPS acoustic space is similar to the acoustic dimension separating discomfort cries from pain cries may seem surprising. Rather, the acoustic dimensions coding for static information (individual signatures) was expected to differ from those coding for dynamic information (distress level). This result suggests two hypotheses. Firstly, the strength of individual signatures in discomfort cries, illustrated by the wide dispersion of babies on the PC1 may reflect small variation in the morphology of the vocal apparatus (larynx and vocal tract) of each individual. A second hypothesis is that not all babies are similarly affected by our mild discomfort (bathing) condition, creating *de facto* acoustic differences between individuals in this context (see, for example, our discussion of VIC). To test this second hypothesis, it would be necessary to standardize the discomfort situation to a greater extent, although this is not guaranteed to be successful, given that there is certainly variability in the way babies react to simple discomfort of any kind. In either case, the variability may be blurred in pain cries as the vocal apparatus is pushed toward the extreme and features specific to distress (e.g., unstable source) take over the available acoustic space.

However, the individual signature carried by discomfort cries is not limited to the PC1 acoustic dimension. Primarily linked to the fundamental frequency of the cry (perceived as the pitch of the voice), the second dimension PC2 also plays an important role. Previous research has shown that this acoustic parameter is an essential marker of sender identity, and that it is highly stable: the pitch of a baby’s discomfort cries predicts the pitch of its voice at the age of 5,^11^ and even in adulthood.^12^ In adults, differences between individuals’ mean fundamental frequencies are preserved between speech types^19^ and in non-verbal vocalizations.^10^ In painful situations, babies experience excitation of the autonomic nervous system and a decrease in their vagal tone.^3^^,^^20^ These changes result in increased tension in the structures of the vocal tract (intrinsic contraction of the larynx and tension of the vocal folds),^21^ which impacts the cry’s fundamental frequency.^20^^,^^21^ Moreover, as a result of irregular or asynchronous vibration of the vocal folds,^22^^,^^23^ the fundamental frequency of the cry as perceived by a listener (i.e., the pitch) is modified by the presence of nonlinear acoustic phenomena characteristic of pain cries (deterministic chaos, biphonation, and subharmonics).^2^^,^^24^ Not only are these nonlinear phenomena inherently less stable and thus likely to fragilize source-related cues to identity, but they can also lead to “pseudoformants” that will also weaken the production and perception of filter-related cues to identity.

The number of false positives (i.e., cries from unknown babies mistakenly attributed to the assigned baby) is an interesting element to consider. In previous studies that also tested recognition of babies by their cries, the proportion of false positives was around 20%. In other words, when an adult hears a cry of discomfort from an unknown baby, they declare it to be their baby around 1 in 5 times (17% in parents^14^ and 23% in non-parents^13^ tested with cries of discomfort; 21% in mothers tested with cries of hunger^15^; and 25% in mothers tested with a mix of cries of hunger and pain^25^). Our results show that the proportion of false positives rises to 30% when a baby is identified solely based on pain cries. The loss of individual information in pain cries therefore accompanies an increase in false positives. Identifying stranger babies as own when in doubt gives a margin of safety to the baby-caregiver communication system, reducing the risk of failing to respond in a situation when promptness to respond is paramount to the baby’s survival. This communication trade-off, in which the ability to identify a baby decreases with increasing distress, could represent an ecological advantage. For human babies, any potential caregiver, parent or non-parent, will essentially receive “pain” information, and this will be little noised by the baby’s “individual identity” information. In the context of the cooperative breeding characteristic of the human species,^26^ this may encourage cooperation and as it fits in with our social functioning, no particular pressure prevents it from working. In addition, soliciting help from any protective in-group caregiver results in more chance that a caregiver will come and help in such urgency case, which might promote babies’ fitness and survival. It is interesting to note that in a non-human mammal, the yellow-bellied marmot *Marmota flaviventris*, which lives in loose social groups and where cooperative breeding is facultative, the pup’s screams elicited by high distress remain highly individualized compared to lower risk alarm calls and induce a specific response from the mothers.^27^^,^^28^

From a methodological point of view, our study illustrates the interest of considering a holistic description of the acoustic structure of signals using MPS rather than a description by discrete variables, when we are interested in the quantity of information carried by signals. While modulation power spectra may be more difficult to interpret than predefined acoustic parameters, they allow capturing information carried by joined spectral-temporal modulations that might be missed in predefined acoustic parameters.^29^ As a result, classifier results are better when they work based on modulation spectra than when they are based on a set of predefined parameters. In addition, using the MPS allows calculating a vector difference between two sounds corresponding to an acoustic distance in a metric space. Distances using a vector of PAF measures require additional orthogonalization and result in a weighted acoustic space, which depends on the correlations found in the sound ensemble being analyzed.

In conclusion, the present study shows that static information (vocal signatures) and dynamic information (stress level) compete in babies’ cries as they are not encoded by fully independent acoustic features. Indeed, the emergence of distress-induced information in pain cries is at the expense of static cues to identity. While this may result from biomechanical constraints affecting sound production by a highly aroused, tensed vocal apparatus, it also makes sense from a survival perspective to favor the communication of urgency over identity in high distress contexts. By becoming less identifiable, a baby in pain may increase its chances of being rescued by any potential caregiver, related or not, which might have promoted fitness more than a personal signature calling the attention only from a parental figure. Moreover, in humans, cooperative breeding may have relaxed selection pressure on encoding identity and instead favored the encoding of dynamic information, especially in emergency contexts. Future studies should investigate the extent to which cue to pain interfere with cues identity in a range of species with different breeding systems.

### Limitations of the study

Our study has several limitations. The first is the limited number of pain cries available for each baby (only 4 pain cries per individual). Pain cries were recorded during vaccine injections, and it was not possible to induce them over a prolonged period. This situation probably mirrors what happens in real life, where it is rare for a baby to cry in pain, whereas cries of discomfort are the most common. In real life, therefore, parents or other caregivers have little opportunity to learn an individual signature from pain cries. The fact that these cries of pain are not very individualized thus should not interfere with learning to recognize this individual signature, as pain cries remain rare.

For both classifier training and playbacks with human participants, we considered only babies for whom we had at least 5 different discomfort calls for the training set. This avoided over-fitting the classifiers and the opportunity for participants to learn. However, the limited number of pain cries per baby allowed only a partial representation of the range of cries potentially emitted by each baby. It is possible, for example, that cries emitted in a more painful context would be even less individually signed. It is however important to note that pain cries are typically rare, thus giving little opportunity for caregivers to learn the voice of individual babies during their development. From this perspective, it appears adaptive to favor the expression of distress and urgency in these contexts.

A second limitation is that participants only completed a short training session (10 short sequences of discomfort cries). This degree of exposure to cries is undoubtedly much less than that experienced by parents in everyday life, where learning opportunities from their baby’s cries are frequent and repeated. Our experimental protocol, in which participants listen to an assigned baby’s cries from headphones, is also far from natural conditions where the baby’s physical presence and the attachment bonds they develop with their caregivers certainly influence learning to recognize their cries.

## STAR★Methods

### Key resources table

**Table:** 

REAGENT or RESOURCE	SOURCE	IDENTIFIER
**Deposited data**		

Acoustic signals	Koutseff et al.^2^	https://doi.org/10.1080/09524622.2017.1344931
Dataset, acoustic signals and statistical codes	This article	https://doi.org/10.5281/zenodo.11403981

**Software and algorithms**		

R	R Project	RRID:SCR_001905 https://www.r-project.org/
BioSound Python package		https://doi.org/10.5281/zenodo.11061994
Labvanced	Finger et al.^30^	https://www.labvanced.com/

### Resource availability

#### Lead contact

Further information and requests for resources and reagents should be directed to and will be fulfilled by the lead contact, Nicolas Mathevon (mathevon@univ-st-etienne.fr).

#### Materials availability

This study did not generate new unique reagents.

#### Data and code availability

• Dataset have been deposited on GitHub and are publicly available as of the date of publication.^31^ The DOI is listed in the key resources table.
• The R code used to analyze the human subject’s classification performance has been deposited on GitHub and is publicly available as of the publication date.^31^ The DOI is listed in the key resources table. The BioSound Python libraries used to extract the bio acoustical features (PAFs and MPS) and perform supervised classification are publicly available at Github (https://github.com/theunissenlab/soundsig) and includes with jupyter notebooks as tutorials (https://github.com/theunissenlab/BioSoundTutorial).^32^ The custom code that uses BioSound to perform the acoustical and corresponding clustering and statistical analyses on this data set has been packaged as a separate set of jupyter notebooks.^31^
• Any additional information required to reanalyze the data reported in this paper is available from the lead contact upon request.

### Experimental model and study participant details

A total of 120 participants were recruited through the Prolific online platform (www.prolific.co/). Once recruited, participants were redirected to the Labvanced online platform (https://www.labvanced.com/)^30^ where the experiment was hosted. After completing the experiment, subjects were redirected to Prolific and paid at the recommended rate of 7.5 GBP per hour. All self-reported to have normal hearing.

Sixteen of them dropped out before the testing session, giving data for 104 participants. To ensure data quality in the context of online experiments, all submissions were manually screened for obvious cheating. Data from four participants were excluded because of abnormally long reaction times, always responding the same, or launching copies of the experiment leading to training on both assigned and stranger babies’ cries. Analysis were therefore done on 100 participants, who were distributed as follows:

- 50 participants were non-parents (N = 25 men, 25 women, mean age = 23.1 ± 4.2 years);
- 50 participants were parents of at least one child younger than 2 years old (N = 25 fathers, 25 mothers, mean age = 33.5 ± 4.9 years).

All participants lived in Europe. The majority of participants (90 out of 100) were Europeans. The remaining 10 participants were from various countries (North America, South America, Africa, Middle East). Due to French legislation, no data on race or ethnicity was collected.

The local ethics committee approved the experiment (October 2019 – Comité d’Ethique du CHU de Saint-Etienne, Institutional Review Board: IORG0007394), and informed consent was obtained from all participants.

### Method details

#### Sound recordings

Cry recordings were extracted from a cry database previously built by the ENES Laboratory.^2^ Discomfort cries were recorded during bathing, undressing, or dressing by parents at the baby’s home. Pain cries were recorded during scheduled routine vaccination at the doctor’s office (N = 22 babies, 10 boys and 12 girls; age = 60.3 ± 3.4 days on the vaccination day; delay between recording sessions of discomfort cries and pain cries for a given baby = 6.9 ± 3 days, see^2^ for a complete description of the recording parameters).

Using Audacity software (www.audacityteam.org), we isolated 284 cry sequences from these raw recordings (mean sequence duration = 6.3 ± 1.1 s; 4 sequences of pain cry per baby and 2-19 sequences of discomfort cry per baby). We took care to isolate sequences without background noise such as adult voices, water flowing or door slamming.

The sound intensity of the cry sequences was normalized to 100% of the maximal amplitude using the “normalize” function of the R package *tuneR*. These sequences were used as stimuli during the experiment, and are referred to as “discomfort cries” and “pain cries”.

#### Acoustic analyses

A total of 22 predefined acoustic features (22 PAFs) are extracted from the measures obtained from the amplitude envelope (mean, sd, skew, kurtosis, entropy, rms, max), the power spectrum (mean, sd, skew, kurtosis, entropy, Q1, Q2 or median, Q3), the time-varying fundamental (meanF0, MaxF0, MinF0, cvF0, mean pitch-saliency) and the average frequency of the first two formants (F1, F2). The time-varying pitch saliency quantifies the strength of the periodicity in the signal and is defined by the relative amplitude of the first peak in the auto-correlation function of the signal obtained in a time-running window of 33 ms. See Elie and Theunissen^33^ for additional details on how these values are calculated. In some analyses we restricted the 22 PAFs to a subset of 10 capturing the fundamental and spectral features (Q1, Q2, Q3, meanF0, MaxF0, MinF0, cvF0, mean pitch-saliency, F1, F2).

The modulation power spectrum (MPS) is obtained by calculating and averaging the amplitude of the 2D Fourier Transform (FT) of log of the spectrogram in 1s (1s = 6sd) gaussian windows. The spectrogram was calculated using a gaussian window with a width in the frequency dimension set at 50 Hz (50Hz = 6sd), in what is often referred to as a narrow-band spectrogram. Before taking the 2D FT the log spectrogram of each baby cry by normalized by its maximum dB value and, after this normalization, minimum values were bounded at -50 dB. The MPS estimated from this normalized log spectrogram is insensitive to the overall rms of the sound and simply captures the overall presence of structure characterized by relative power in specific joint spectral-temporal modulations. Because the MPS is highly dimensional, it was projected to a subspace determined by its first 20 principal components (PCs). These PCs are equivalent to the linear discriminant functions that describe the best acoustical subspace for discriminating baby identity, assuming equal and diagonal covariance matrix. The first 2 PCs captured 55% of this between baby MPS variance in the discomfort cries. The first PC (PC1) captures a spread of the spectral-temporal modulations around the central pitch region and is strikingly similar to the difference in average MPS observed between discomfort and pain (compare Figure 2B, PC1, with Figure 1C, Pain-Discomfort). The second PC (PC2) captures a sharpening of the central pitch region associated with a more stable pitch (i.e. continuous) at the corresponding fundamental frequency. The inhibitory (blue) band shown for lower spectral modulations will also result in higher PC2 coefficient values for cries that lack power in these lower frequency modulations, which correspond to higher values of the pitch fundamental.

All the acoustic parameters were estimated using the BioSound python package (https://github.com/theunissenlab/BioSoundTutorial).

To visualize the individual signature of the baby cries and how this signature changes in the two conditions, we plotted the average MPS represented by its first two principal component coefficients (PC1, PC1) in the two conditions. To quantify this reduction in individual signature, we estimated the distance of each MPS to the average across babies in this 2D acoustic space. We repeated the analysis but by calculating the PCs in the Pain condition instead of the discomfort condition and this finding acoustic dimensions optimized to separate babies from their cries in the Pain condition.

#### Supervised classification

Linear Discriminant Analysis (LDA) was used to assess the discriminability of the baby’s identity based on acoustic features. Eighteen babies for which we had at least 5 samples of discomfort cries for the training set were used. When tested within-condition, we used a leave one out cross-validation (LOO) and therefore all the babies used in these analyses had at least 6 samples of discomfort cries. We also required all babies to have at least 4 samples of pain cries and, thus, a minimum of ten exemplars of cries per baby. Posterior probabilities obtained in cross-validation were represented with a confusion matrix. This LDA was done using either the 22 predefined acoustic features (PAFs), limited to a subset of 10 spectral features, or the Modulation Power Spectrum (MPS). We reported the number of correctly classified stimuli in cross-validation. Cross-validation was performed using LOO when the fitting and test set of baby cries were taken from the same condition(s) (i.e. discomfort and/or pain). When the fitting and testing set were from different conditions, here discomfort and pain respectively, we used all the data in the discomfort condition for fitting and all the data available in the pain condition for testing in a single simple cross-validation. Analyses were repeated using Quadratic discriminant analysis (QDA) and Random Forest Classifiers (RF). LDA, QDA and RF were performed using Python scikit-learn package (1.2.2). Additional custom python code was used to perform the cross-validation and, as needed, dimensionality reduction of the feature space to ensure robustness. This supervised classification code is also part of the BioSound package. Binomial exact tests were performed to directly compare the number of baby cries in the cross-validation set whose identity was correctly identified to the number expected by chance given the total sample size. Fisher exact tests were used to compare classification performance under different conditions based on the number of babies correctly classified and the sample size in each condition.

#### Psychoacoustic experiment

The experimental procedure included two successive training sessions over two days followed by a test session a few hours later^5^(mean delay from the end of the second training session to the beginning of the test session = 6.4 ± 4.4 h, min-max = 3-22.6). During the training sessions, participants listened to discomfort cries from their assigned, “familiar” baby (5 different cries per session; different cries between sessions). During the test session, participants listened to 20 different pain cries, including 4 pain cries from their “familiar” baby and 4 pain cries from 4 unknown babies (2 boys, 2 girls), presented in random order.

The discomfort cries used as stimuli in the training sessions came from the 9 babies (3 boys, 6 girls) for whom we could extract at least 10 discomfort cry sequences. To diversify the identity of the babies, the pain cries used as stimuli in the test session were extracted from the recordings of the 22 babies (i.e. from the complete databank). Randomization of stimuli among the tested listeners was performed using an R script implementing the “sample” function.

At the beginning of each training session, participants were given the following instruction: “You are going to hear several cries of one baby, who will be named YOUR baby for the rest of this study”. Then, the participants’ task during the test session was to decide whether each cry they heard was produced by their “familiar” baby or by an unknown baby. The playback experiment was designed to elicit 20% “yes” responses (4 out of 20 cries from the familiar baby) and 80% “no” responses (16 out of 20 cries from unknown babies). Participants were unaware that the cries were from different contexts (discomfort cries for training and pain cries for testing). To avoid decision bias, participants did not know how many cries came from their assigned baby or how many different babies they were listening to.

### Quantification and statistical analysis

#### Acoustic features

We used mixed-effect statistical modeling to assess differences in acoustical features between cries produced either in the discomfort or pain condition. The response variable was the acoustic feature. The fixed effects were the baby’s sex and condition and their interaction, and the random effect was the intercept for each baby. We used Wald test to assess whether the fixed effects’ coefficients differed from zero. The mixed effects statistical modeling was performed using the mixedlm() function from the Python package *statsmodels* (v0.13.5).

#### Psychoacoustic experiment

Statistical analyses for analyzing the psychoacoustical data were performed in R (v4.2.2) using Bayesian mixed models fitted with the brms R package.^34^ The advantages of using the Bayesian approach are multiple, including its high flexibility, quantification of uncertainty in estimates, the use of priors for regularization and intuitive interpretation of confidence intervals.^35^^,^^36^ Response scores were modeled using a logistic function (Bernoulli family). The outcome variable was the binary “yes/no” response that participants gave when asked whether or not the cry belonged to their assigned baby. The baby status (familiar or unknown), participants’ sex and parental status (parent or non-parent) were included in the model as fixed factors. Participant identity and familiar baby identity were included as random factors. The need for random slopes was determined using the Watanabe-Akaike information criterion for model selection (*WAIC*). The structure of the model in R compact notation was: rated_baby_status ∼ real_baby_status x parentality x sex + (real_baby_status + parentality|babyID_ref) + (real_baby_status|subjectID). 3000 iterations were run over two MCMC chains (Markov chain Monte Carlo) with the first 1000 iterations of each chain used to adjust the algorithm. We specified normal priors with mean 0 and a standardized sd = 1 on the coefficients that are used to model effects (slope) and a normal prior with mean given by the chance level (20%) and sd = 1 for the coefficient corresponding to the intercept. In other words, we used priors favoring the Null hypothesis to prevent overfitting. Results were summarized as medians of the posterior distributions and 95% credible intervals (CIs). Credible intervals for estimates above the chance level indicate a credible effect given the observed data and model structure.^35^ Similarly, when contrasting two conditions, CIs excluding the null value can be inferred to indicate a credible difference between the conditions.
