# Supplementary material for: Pain cues override identity cues in baby cries
Source: iScience. 2024 Jun 24;27(7):110375. doi: 10.1016/j.isci.2024.110375 (PMC11269312; doi:10.1016/j.isci.2024.110375)
Supplement: Document S1. Figures S1–S3 and Tables S1 and S2 [file mmc1.pdf]

iScience, Volume *27*

Supplemental information

Pain cues override identity cues in baby cries

Siloé Corvin, Camille Fauchon, Hugues Patural, Roland Peyron, David Reby, Frédéric Theunissen, and Nicolas Mathevon

Supplementary Tables

Type	LDA Correct	QDA Correct	RF Correct	Tested	LDA Pval	QDA Pval	RF Pval
Discomfort	103	61	73	184	$2.02\ 10^{-78}$	$9.70\ 10^{-31}$	$1.26\ 10^{-42}$
Mixed	101	81	101	256	$4.89\ 10^{-58}$	$1.43\ 10^{-38}$	$4.89\ 10^{-58}$
Discomfort/Pain	13	7	14	72	$1.54\ 10^{-04}$	$1.05\ 10^{-01}$	$3.73\ 10^{-05}$
Pain/Pain	17	n.a.	15	72	$3.25\ 10^{-07}$	n.a.	$8.29\ 10^{-6}$

Type	LDA Post	QDA Post	RF Post	LDA Post 2SE	QDA Post 2SE	RF Post 2SE
Discomfort	41.98%	26.42%	24.71%	7.28%	6.50%	6.36%
Mixed	27.50%	28.10%	20.76%	5.58%	5.62%	5.07%
Discomfort/Pain	14.43%	8.34%	12.96%	8.28%	6.52%	7.92%
Pain/Pain	13.62%	n.a.	13.38%	8.08%	n.a.	8.02%

Table S1. Supervised classifier results for correct baby identification based on the Modulation Power Spectrum of their cries, **Related to Figure 2.** Results in cross-validation of the Linear Discriminant Analysis (LDA), the Quadratic discriminant analysis (QDA) and Random Forest Classifiers (RF) with 4 different training/testing sets: Discomfort/Discomfort, Mixed/Mixed or Discomfort/Pain. In the top table, the LDA Pval, etc are the p-values obtained in a binomial test for the observed proportion and larger given the chance level of $p = 1/18 = 0.0556$. The bottom table shows the average posterior probability of correct classification in % and the 2 standard errors (SE) for those estimates. The fact that the performance of the QDA and RF is slightly worse suggests that they are more susceptible to overfitting. The QDA classifier could not be fitted with the pain set because we did not have enough data to fit a within covariance matrix unique to each baby (n.a.: not available).

Type	Features	LDA Correct	QDA Correct	RF Correct	Tested	LDA Pval	QDA Pval	RF Pval
Discomfort/ Discomfort	PAFs	83	67	87	184	$1.30\ 10^{-53}$	$1.68\ 10^{-36}$	$2.84\ 10^{-58}$
Discomfort/ Discomfort	10 Spectral	74	60	84	184	$1.11\ 10^{-43}$	$8.14\ 10^{-30}$	$9.19\ 10^{-55}$
Mixed/ Mixed	PAFs	95	87	104	256	$6.86\ 10^{-52}$	$4.24\ 10^{-44}$	$3.31\ 10^{-61}$
Discomfort/ Pain	PAFs	9	12	11	72	$1.81\ 10^{-2}$	$5.84\ 10^{-04}$	$2.02\ 10^{-3}$
Pain/Pain	PAFs	18	n.a.	14	72	$5.76\ 10^{-8}$	n.a.	$3.73\ 10^{-5}$

Type	Features	LDA Post	QDA Post	RF Post	LDA Post 2SE	QDA Post 2SE	RF Post 2SE
Discomfort/ Discomfort	PAFs	31.90%	27.15%	25.67%	6.87%	6.56%	6.44%
Discomfort/ Discomfort	10 Spectral	30.53%	26.14%	25.65%	6.79%	6.48%	6.44%
Mixed/ Mixed	PAFs	26.05%	30.99%	21.24%	5.49%	5.78%	5.11%
Discomfort/ Pain	PAFs	14.23%	15.73%	12.29%	8.23%	8.58%	7.74%
Pain/Pain	PAFs	14.93%	n.a.	13.28%	8.40%	n.a.	8.00%

Table S2. Supervised classifier results for correct baby identification based on the Predefined Acoustic Features (PAFs or 10 selected spectral features) of their cries, Related to Figure S1. Results of the Linear Discriminant Analysis (LDA), the Quadratic discriminant analysis (QDA) and Random Forest Classifiers (RF), with 4 different training/testing sets: Discomfort/Discomfort, Mixed/Mixed, Discomfort/Pain or Pain/Pain. The QDA classifier could not be fitted with the pain set because we did not have enough data to fit a within covariance matrix unique to each baby (n.a. : not available).

Supplementary Figures

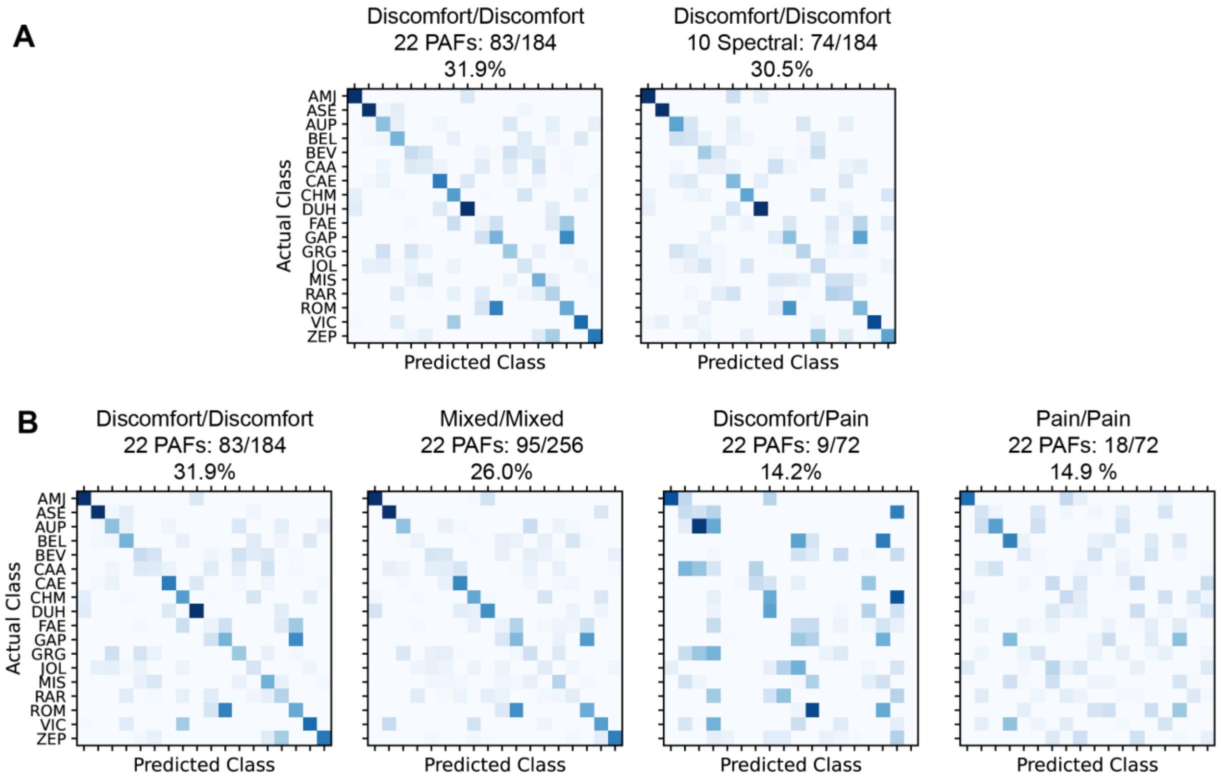

Figure S1. Linear Discriminant Analysis (LDA) Results Based on PAFs, related to Figure 2. LDA was used to assess the discriminability of the baby's identity based on Predefined Acoustic Features (PAFs). Eighteen babies for which we had at least 5 samples of discomfort cries for the training data set were used. Confusion matrices of posterior probabilities obtained in cross-validation are represented. The title above shows the condition of the stimuli used in training/validation. The numbers on the second line show the number of correctly classified stimuli in the cross-validation based on maximum posterior probability. The percent indicated above each confusion matrix is the average of the posterior probabilities for correct classification (found on the diagonal of the confusion matrices). The color scale is the same as in Fig. 2. **A.** Comparison of the performance of classifier in the Discomfort/Discomfort train/test condition when entire set of 22 PAFs are used versus when they are limited to a subset of 10 spectral features. **B.** Comparison of classifier performance based on the 22 PAFs but with different conditions in the training and testing set as in *Figure 2*.

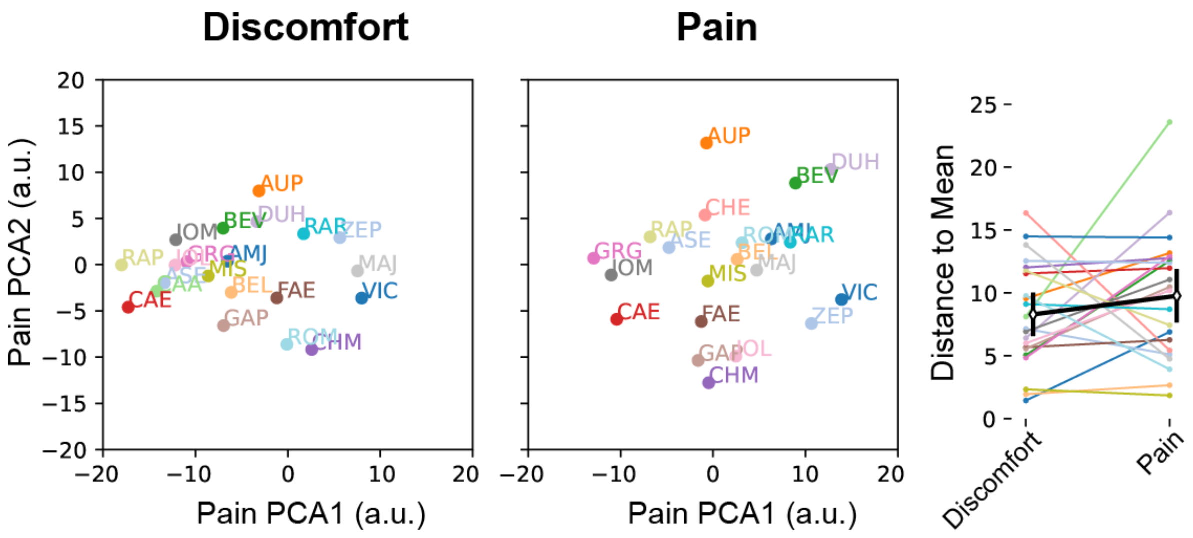

Figure S2. Variability in individual signatures is lower and signatures are not coded differently in the acoustic space of pain cries compared with discomfort condition, Related to Figure 3. We repeated the analysis shown in Fig. 2 by calculating the PCs in the Pain condition instead of the discomfort condition. In this representation, there is no difference in the extend of the spanned acoustic area ($t(21) = -1.141$ $p = 0.2666$).

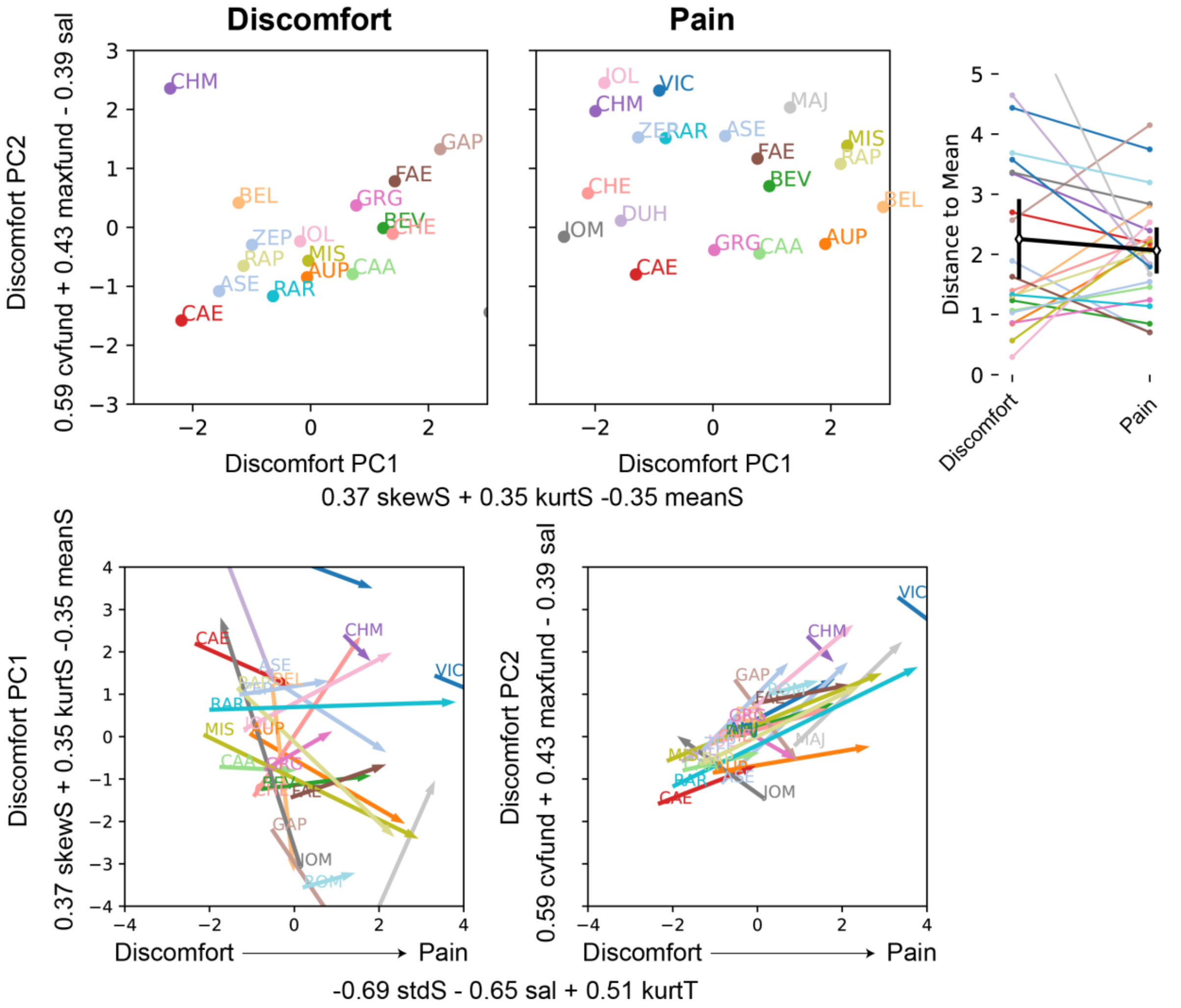

Figure S3. The discomfort-pain dimension also corresponds to a dimension obtained by PCA on PAFs to distinguish baby identities, related to Figure 3. We repeated the analysis shown in Figure 3 but by estimating the principal components (PCs) from the 22 measures of predefined acoustic features. Using this spatial representation, we do not observe the shrinking in the space occupied that was observed with the PCs calculated with the MPS (paired t-test, $t(21) = 0.551$, $p = 0.5875$). On the contrary, the points appeared more randomly scattered (but with similar variance) in the pain condition. Note that contrary to the space obtained from MPS, the space obtained from PAFs is not necessarily metric. Using the PCs obtained from the PAFs, we did however find that one of the major dimensions used for distinguishing the baby identity, here PC2, is highly correlated with the dimension that separates pain from discomfort cries (correlation between the Discomfort-Pain axis and PC2 of the discomfort cries: $r = 0.798$, Fisher's exact test $p = 8.617e\text{-}06$; correlation for the pain cries: $r = 0.702$, $p = 0.00027$; correlation for pair-wise difference discomfort-pain: $r = 0.545$, $p = 0.008678$, N = 22 babies). The PC2 is a dimension that captures variance in parameters that describe pitch saliency (sal) and variability in the fundamental frequency (maxfund, cvfund). PC1, which captures variation in coarse spectral envelope (i.e. skewS, kurtS, meanS; parameters typically associated with formants) is not correlated with the dimension separating discomfort from pain cries (correlation for the discomfort cries: $r = -0.246$, Fisher's exact test $p = 0.2689$; correlation for the pain cries: $r = -0.121$, $p = 0.5915$; correlation for pair-wise difference discomfort-pain: $r = -0.261$, $p = 0.2416$, N = 22 babies).
